# Validation of Urine Test for Detection of* Helicobacter pylori *Infection in Indonesian Population

**DOI:** 10.1155/2015/152823

**Published:** 2015-12-28

**Authors:** Ari Fahrial Syam, Muhammad Miftahussurur, Willy Brodus Uwan, David Simanjuntak, Tomohisa Uchida, Yoshio Yamaoka

**Affiliations:** ^1^Division of Gastroenterology, Department of Internal Medicine, Faculty of Medicine, University of Indonesia, Jakarta 10430, Indonesia; ^2^Department of Environmental and Preventive Medicine, Oita University Faculty of Medicine, Yufu 879-5593, Japan; ^3^Gastroentero-Hepatology Division, Department of Internal Medicine, Airlangga University Faculty of Medicine, Surabaya 60131, Indonesia; ^4^Institute of Tropical Disease, Airlangga University, Surabaya 60115, Indonesia; ^5^Department of Internal Medicine, Santo Antonius Hospital, Pontianak 78115, Indonesia; ^6^Department of Internal Medicine, Yowari Hospital, Jayapura 99352, Indonesia; ^7^Department of Molecular Pathology, Oita University Faculty of Medicine, Yufu 879-5593, Japan; ^8^Department of Gastroenterology and Hepatology, Baylor College of Medicine and Michael E. DeBakey Veterans Affairs Medical Center, Houston, TX 77030, USA

## Abstract

We measured the accuracy of the urine test (RAPIRUN) for detection of* Helicobacter pylori* infection in Indonesia (Jakarta, Pontianak, and Jayapura) using histology confirmed by immunohistochemistry and/or culture as gold standards. We also used immunohistochemistry to identify CagA phenotype and analyzed* H. pylori* CagA diversity in Indonesia. The overall prevalence of* H. pylori* infection in 88 consecutive dyspeptic patients based on the urine test was 15.9% (14/88), 38.1% for patients in Jayapura that had higher prevalence of* H. pylori* infection than that in Jakarta (9.7%, *P* = 0.02) and Pontianak (8.3%, *P* = 0.006). Overall sensitivity, specificity, positive predictive value, negative predictive value, and accuracy of RAPIRUN were 83.3%, 94.7%, 71.4%, 97.3%, and 93.2%, respectively. All of the* H. pylori-*positive patients were immunoreactive for anti-CagA antibody but not immunoreactive for East Asian specific anti-CagA antibody in all* H. pylori-*positive subjects. We confirmed the high accuracy of RAPIRUN in Indonesian population. In general, we found less virulent type of* H. pylori* in Indonesia, which partly explained the low incidence gastric cancer in Indonesia.

## 1. Introduction

*Helicobacter pylori* infection is regarded as a high risk factor for severe gastritis associated diseases, including peptic ulcers and gastric cancer [1]. Although* H. pylori* was discovered more than 30 years ago by Marshall and Warren [2], it is still debatable which methods can be considered as a gold standard for detection of* H. pylori* infection. Recently several direct diagnostic tests including histopathology and/or immunohistochemistry (IHC), rapid urease test, and culture are frequently used due to the ability to obtain genotype and antibiotic resistance information. However due to the fact that small amount of bacteria that colonize the stomach in these clinical circumstances leads to decreased sensitivity of the direct tests, several indirect tests including antibody-based test including serology and urine test, urea breath test, and stool antigen test have been developed to diagnose* H. pylori* infection [3].

Indonesia is a developing country at the southeastern tip of mainland Asia and Oceania; it is an archipelago with a multiethnic society with more than 1,000 ethnic and subethnic groups. The age-standardized incidence rate of gastric cancer in Indonesia was reported to be 2.8/100,000, which is relatively low among Asian countries (available from the International Agency for Research on Cancer; GLOBOCAN2012, http://globocan.iarc.fr/). In our previous study using five different tests, the prevalence of* H. pylori* infection was only 11.5% in Surabaya in Java island, Indonesia [4]. Until March 2013, only 313 hospitals were currently providing GI endoscopy services in Indonesia. Although it is distributed in 33 provinces around the country, 72% (98/136) of them are on Java island [5]. Moreover, many patients with dyspepsia are not covered by the Indonesian health insurance system so as it is difficult for them to undergo endoscopy. Therefore, although the invasive method gives more information, the indirect methods are the best choice for measuring* H. pylori* infection in the lacking of endoscopy system area, and the rapid urine test becomes one option.

Several studies reported the presence of antibody to* H. pylori* in body fluids other than serum including saliva and urine [6–9]. In addition, urine can be obtained easily and its collection requires few skills and does not require centrifugation, and urine-based test is cheaper than that of serum [10]. A urine-based rapid test kit, RAPIRUN* H. pylori* antibody (RAPIRUN), was developed by Otsuka Pharmaceutical Co., Ltd. (Tokyo, Japan) for detection of antibody to* H. pylori* in urine. The accuracy of RAPIRUN has been reported to be high with excellent sensitivity, specificity, and accuracy for Japanese population (92.0%, 93.1%, and 92.3%, resp.) [11] as well as for Vietnamese population [12]. In 2011, the revised stick-type of RAPIRUN (RAPIRUN Stick) was introduced to have higher agreement rate (98.4%) compared with the conventional RAPIRUN in the Japanese population with a shorter time (15 min versus 20 min) [13]. However RAPIRUN developed based on a Japanese* H. pylori* strain (OHPC-040 strain) [9]. Therefore, it needed validation in Indonesian population. Moreover, all guidelines recommended using only validated commercial tests [14–19]. In this study, we measured the accuracy of the urine test RAPIRUN in Indonesia population using histology confirmed IHC and culture as a gold standard.

On the other hand, several studies have shown that IHC staining with specific* H. pylori* antibodies has the highest sensitivity and specificity and better interobserver agreement compared to histochemical stains [20]. Recently, we also successfully generated an anti-East Asian type CagA-specific antibody (*α*-EAS Ab) which was immunoreactive only with the East Asian type CagA and not with the Western CagA [21]. We have also shown that *α*-EAS Ab was a useful tool for typing CagA immunohistochemically in Japanese [22] and Vietnamese and Thai [23] people, with the sensitivity, specificity, and accuracy of 93.2%, 72.7%, and 91.6% and 96.7%, 97.9%, and 97.1%, respectively. For the second purpose of this study, we used IHC to identify CagA phenotype and analyzed influence of* H. pylori* CagA diversity on gastric mucosal status in Indonesia.

## 2. Methods

### 2.1. Study Population and* H. pylori* Infection Status

**Figure 1:**
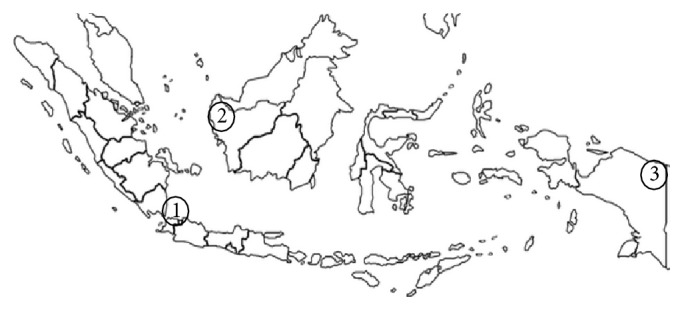
Collecting areas. Urine was collected from the (1) Jakarta (Java island), (2) Pontianak (Borneo island), and (3) Jayapura (Papua island).

We performed prospective study from January 2014 to September 2014. The survey took place on Jakarta (*n* = 31) in Java island, Jayapura (*n* = 21) in Papua island, and Pontianak (*n* = 36) in Borneo island (Figure 1). Experienced endoscopists (AS and WU) collected three gastric biopsy specimens from consecutive dyspeptic patients during each endoscopy session: two samples from the lesser curvature of the antrum approximately 3 cm from the pyloric ring (culture and histology) and one sample from the greater curvature of the corpus (histology). Biopsy specimens for culture were immediately placed at −20°C and stored at −80°C within a day of collection until they were used for culture testing. We excluded patients with the history of partial gastric resection and received* H. pylori* eradication. We also obtained information about medications (e.g., nonsteroidal anti-inflammatory drugs, low-doses of aspirin, antibiotics, histamine-2 receptor antagonists, or proton pump inhibitors). We excluded patients with regular use of nonsteroidal anti-inflammatory drugs and low-doses of aspirin and also those with any antibiotics, histamine-2 receptor antagonists, or proton pump inhibitors for the previous 4 weeks. To minimize the potential bias, we used the same experienced pathologist (TU) that performed the experiments, who also performed experiments in Myanmar, Vietnam, Bhutan, Dominican Republic, and Indonesia [4, 24–28]. Informed consent was obtained from all participants, and the protocol was approved by the Ethics Committee of Dr. Cipto Mangunkusumo Teaching Hospital (Jakarta, Indonesia) and Oita University Faculty of Medicine (Yufu, Japan).

### 2.2. Rapid Urinary Test

All urine samples were measured and analyzed while being blinded to subjects' information. Urinary* H. pylori* antibody status was determined with a rapid urine test (RAPIRUN Stick, Otsuka Pharmaceutical Co.). Immediately after collection, urine samples were tested for* H. pylori* antibodies. To perform the test, 0.3 mL of fresh urine was mixed with 0.3 mL of dilute solution using a pipette supplied with the “2-fold dilution.” Then, the test stick was put into the container that holds the mixture of urine and diluent. The test stick contains colloidal gold-conjugated anti-human IgG (Fc) polyclonal antibody (goat). The test line and control line in the evaluation section of the stick are immobilized with* H. pylori* antigen and anti-human IgG polyclonal antibody, respectively [29]. The sample was considered positive when two red bands at the test line and the control line were observed within 15 min at room temperature (25°C–30°C) and negative when only the control line was observed. The absence of a control line indicates an invalid result possibly due to errors in the assay procedure or extremely diluted urine.

### 2.3. Histology and Immunohistochemistry

All biopsy materials for histological testing were fixed in 10% buffered formalin and embedded in paraffin. Serial sections were stained with May-Giemsa stain as well as hematoxylin and eosin. IHC was performed as previously described [22]. After inactivation of endogenous peroxidase activity and antigen retrieval, tissue sections were incubated with *α*-*H. pylori* antibody (DAKO, Denmark), anti-CagA antibody (b-300 Santa Cruz, USA) or *α*-EAS Ab diluted overnight with at 4°C with a comparison 1 : 2,000 with diluting solution (DAKO, Denmark). On the next step, the sections were incubated with biotinylated goat anti-rabbit or anti-rat IgG (Nichirei Co., Japan) and continued by incubation with a solution of avidin-conjugated horseradish peroxidase (Vectastain Elite ABC kit, Vector Laboratories Inc., Burlingame, CA, USA). The peroxidase activity was detected using hydrogen peroxide or diaminobenzidine substrate solution. Positive Giemsa staining for bacteria and positive result of anti-*H. pylori* antibody immunostaining with bacterial loads greater than or equal to grade 1 were considered positive for* H. pylori*.

### 2.4. *H. pylori* Isolation

*H. pylori* colonies were cultured from antral biopsy specimens using standard methods. For* H. pylori* culture, one antral biopsy specimen was homogenized in saline and those inoculated onto Skirrow's medium were incubated for up to 10 days at 37°C under microaerophilic conditions (10% O_2_, 5% CO_2_, and 85% N_2_).* H. pylori* were identified on the basis of colony morphology, Gram staining results, and positive reactions for oxidase, catalase, and urease. Isolated strains were stored at −80°C in Brucella Broth (Difco, NJ, USA) containing 10% dimethylsulfoxide and 10% horse serum. If histology confirmed by IHC and/or culture yielded the positive results for* H. pylori* infection, we regarded the subjects as infected with* H. pylori*.

### 2.5. Statistical Analysis

All data was analyzed using SPSS, version 19 (SPSS Inc., Chicago, IL, USA). Discrete variables were tested using the chi-square test and continuous variables were tested using *t*-tests and Mann-Whitney *U* test. A two-tailed *P* value <0.05 was considered as statistically significant.

## 3. Results

### 3.1. *H. pylori* Infection Rate Using Urine Test

**Figure 2:**
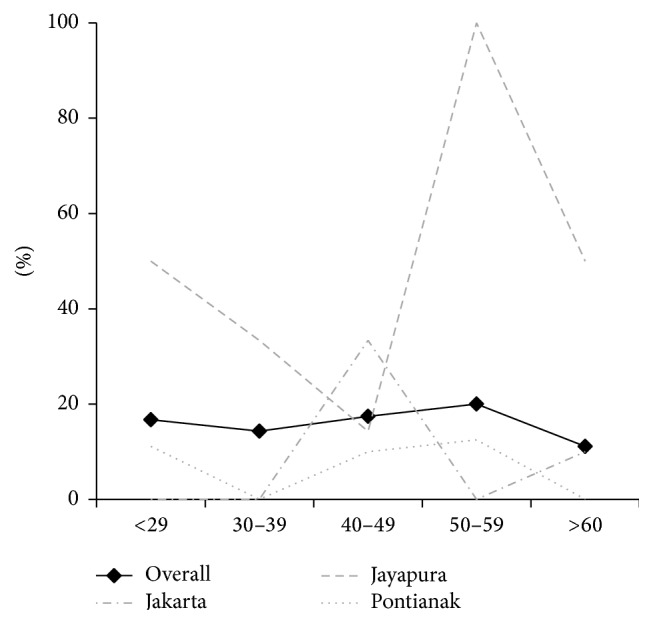
Prevalence of* Helicobacter pylori* infection by age group. Subjects were considered to be* H. pylori*-positive when they showed positive results for the rapid urine test (RAPIRUN* H. pylori* antibody, Otsuka Pharmaceutical Co., Tokyo, Japan). The gray colour describes the prevalence of* H. pylori* in the three different populations.

The total study population was 88 consecutive dyspeptic patients (41 females and 47 males; mean age of 44.7 ± 14.5 years; range, 18–77 years) and consisted of 18 subjects aged ≤29 years, 14 subjects aged 30–39 years, 23 subjects aged 40–49 years, 15 subjects aged 50–59 years, and 18 aged ≥60 years. The overall prevalence of* H. pylori* from three cities based on the urine test was 15.9% (14/88), 16.7% (3/18) aged ≤29 years, 14.3% (2/14) aged 30–39 years, 17.4% (4/23) aged 40–49 years, 20% (3/15) being 50–59 years old, and 11.1% (2/18) being ≥60 years old (Figure 2). There was no relationship between the prevalence of* H. pylori* infection with age and sex (*P* = 0.40 and *P* = 0.28). The patients living in Jayapura (38.1%, 8/21) had higher prevalence of* H. pylori* infection than those in Jakarta (9.7%, 3/31; *P* = 0.02) and Pontianak (8.3%, 3/36; *P* = 0.006). The prevalence of* H. pylori* in the three different populations by age group is shown in Figure 2. There was no statistically significant relationship between* H. pylori* infection rate and history of drugs (*P* > 0.05).

### 3.2. The Accuracy of Urine Test

**Figure 3:**
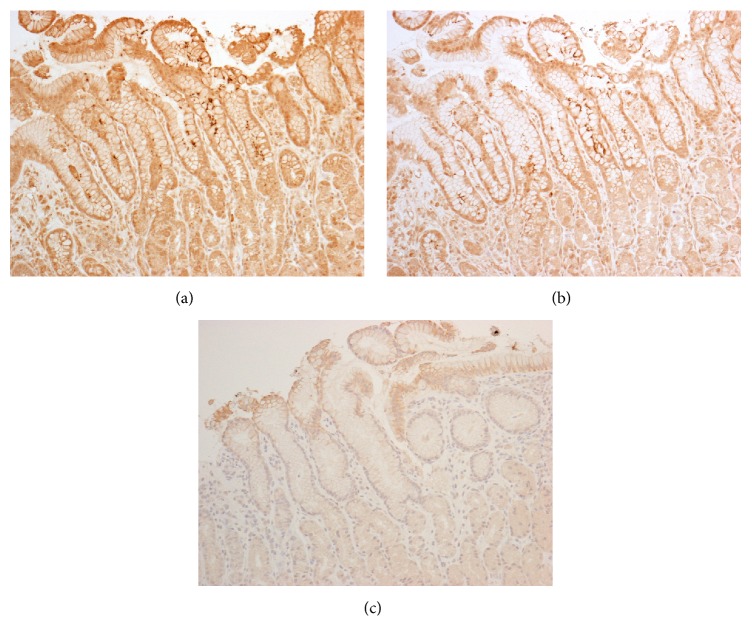
Urine test validation and genotyping* H. pylori* using immunohistochemistry. Gastric mucosa biopsy specimen of a urine test positive patient (Jay4) was positively immunostained with anti-*H. pylori* antibody (a) and anti-CagA antibody (b), but it was not immunostained with anti-East Asian specific antibody (c).

To confirm the accuracy of the urine test, we compared the results of urine test with histology confirmed by IHC (Figure 3(a)) and culture. We found identical results between histology confirmed by IHC and culture (12/88, 13.6%). Only two samples were positive by urine test but negative in both of the gold standards. On the other hand, four patients were negative by urine test but positive with histology confirmed IHC and culture. Overall sensitivity and specificity of RAPIRUN were 83.3% and 94.7%, respectively. Positive predictive value was 71.4% and negative predictive value was 97.3% and the overall accuracy rate was 93.2%.

### 3.3. *H. pylori* Genotypes

All of the* H. pylori-*positive patients by histology that enrolled in this study were immunoreactive for the anti-CagA antibody (Figure 3(b)). Interestingly there were no immunoreactive patients for *α*-EAS Ab in all* H. pylori-*positive ones (Figure 3(c)). The subjects negative for *α*-EAS Ab were regarded as infected with non-East Asian type CagA.

## 4. Discussion

We confirmed the high accuracy of RAPIRUN, the rapid immunochromatographic method for determination of anti-*H. pylori* IgG in urine. This urine test will be reliable to use for detection of* H. pylori* in Indonesia. This result also is in concord with our previous report in North Sulawesi; using the similar kit, the results of urine test were identical with that of anti-*H. pylori* antibody serum test [30]. As a noninvasive test, the urine test is user friendly and of low cost with high accuracy; it is therefore the best option for measuring* H. pylori* status in remote area lacking the endoscopy system in Indonesia. In addition, it would also be very useful for mass screening. The accuracy of RAPIRUN has been reported to be high with excellent sensitivity and specificity: 95.3%, 96.7%; 96.9%, 92.9%; 79.5%, 90.7%, for white, black and Asian [11], and Japanese [31] as well as for Vietnamese population [12], respectively. One recent study in Vietnamese population [29] reported that RAPIRUN Stick showed a better sensitivity (84.7%). The false negative (4.5%) in this study probably related to the low level of urinary anti-*H. pylori* IgG and/or different genotype of* H. pylori*. In contrast, the prolonged positive urine test results after eradication of* H. pylori* infection would be associated with false positive result [11].

In this study, we used histology confirmed IHC and culture as a gold standard. Several guidelines indicate that not one single test can be considered to be the gold standard for diagnosis of* H. pylori* infection and that the suitable test should be chosen after considering the advantages and disadvantages of several tests [14, 16, 18, 19]. Although urea breath test (UBT) and stool antigen test (SAT) are the best methods to determine an active infection among noninvasive tests [14], both tests need a local test validation in order to find the best cut-off for each population [32, 33]. To our knowledge, there has been no consensus which determined the best cut-off of UBT and SAT for Indonesia population. On the other hand, culture remains a reference method as it allows the direct detection of* H. pylori* organisms even though it presents a limited sensitivity. Histology confirmed with IHC could be covering the disadvantages of culture. By adding IHC, we could assess the presence of* H. pylori* with more certainty, especially if inflammation is present or the coccoid forms of* H. pylori* (mimic bacteria or cell debris) caused difficulties to identify by standard staining. Moreover, IHC might be a useful tool for genotyping* H. pylori* without individual bias [34, 35].

The* H. pylori* routes of transmission are still not fully understood. Human-to-human spread through fecal-oral or oral-oral routes is considered to be the most plausible routes for infection [36]. In developing countries,* H. pylori* infection is transmitted mainly through fecal-oral route, whereas in developed countries gastrooral route is usual [37]. Lower social economic status, nonfiltered water, and smoking are risk factor for* H. pylori* [38]. On the other hand the improvement of hygiene conditions has significantly decreased the prevalence of* H. pylori* infection in Europe and North America [39]. Further studies will be necessary to clarify why the prevalence of this infection in patients who are living in Jayapura was higher than two other populations. However generally sanitary conditions are better in Western regions than in eastern areas, although sanitary conditions vary by area in Indonesia. We should also make a count of host genetic factors which might contribute to a reduced susceptibility to* H. pylori* infection, a possibility suggested in the ethnic Malaysian population [40]. The immunoreactive patients for anti-CagA antibody in all* H. pylori*-positive patients were not followed by immunoreactive *α*-EAS Ab. It was indicated that patients were infected with non-East Asian type CagA. Several studies reported that East Asian type CagA has a higher binding affinity for the Src homology-2 domain-containing phosphatase 2 (SHP2), resulting in having high risk of peptic ulcer and/or gastric cancer than Western type CagA [41–44]. Our results suggested that, in addition to host and environmental factors, the low incidence of gastric cancer in Indonesia might be associated with the low prevalence of* H. pylori* infection and less virulent type of* H. pylori* in Indonesia. The small number samples certainly become the limitation in this study. Further studies with increased sample numbers are necessary to better elucidate the main reason of low incidence of gastric cancer in Indonesia.

## 5. Conclusion

We confirmed the high accuracy of RAPIRUN in Indonesian population. We also revealed that the prevalence of* H. pylori* infection in patients who are living in Jayapura is higher than that in Jakarta and Pontianak. However in general we found less virulent type of* H. pylori* in Indonesia, which is partly explained by the low incidence gastric cancer in Indonesia.
